# The effectiveness of double incision technique in uterus preserving surgery for placenta percreta

**DOI:** 10.1186/s12884-017-1262-3

**Published:** 2017-04-27

**Authors:** Ibrahim Polat, Burak Yücel, Ali Gedikbasi, Halil Aslan, Aysun Fendal

**Affiliations:** Department of Obstetrics and Gynecology, Kanuni Sultan Suleyman Training and Research Hospital, Istanbul, Turkey

**Keywords:** Placenta percreta, Conservative surgery, Uterus preserving surgery, Trans-placental approach

## Abstract

**Background:**

Placenta percreta is a life-threatening condition that places patients at risk of massive bleeding. It necessitates very complicated surgery and can result in mortality. Caesarean hysterectomy is the accepted procedure worldwide; however, recent studies discussing conservative treatment with segmental resections have been published. Foetal extraction and segmental resection can be performed through the same incision (single uterine incision) or through two different incisions (double uterine incision). In this study, we aimed to evaluate the effectiveness and results of the double incision technique.

**Methods:**

Twenty-two patients with a diagnosis of placenta percreta who underwent conservative surgery were included. Segmental resection was performed via single incision in ten patients and double incision in twelve patients.

**Results:**

There was no difference between the patients who underwent segmental resection via single and double incision in terms of age, gravida, number of previous caesarean deliveries, gestational age at delivery, or rate of elective surgeries. The operation time, transfusion requirement, intensive care unit admission, total hospitalization and success of conservative surgery were comparable between the groups.

**Conclusions:**

Based on the outcomes of our study, double uterine incision allows for the safe extraction of the foetus during uterus-preserving surgery in patients with placenta percreta without worsening the results compared to single uterine incision.

**Trial registration:**

NCT02702024, Date of registration: February 26, 2016, retrospectively registered.

## Background

The presence of an abnormally implanted, invasive, or adhered placenta is called accrete syndrome (AS) [1]. The causes of this phenomenon are the partial or total absence of the decidua basalis and defective development of the fibrinoid layer. Moreover, if the fibrinoid layer is completely lacking and the physiological line of cleavage is absent, cotyledons are more densely anchored to the myometrium. The surface area of the implantation site involved and the depth of trophoblastic tissue ingrowth are variable among women, but all affected placentas can potentially have significant haemorrhage [2].

The incidence of AS has increased remarkably with the increasing prevalence of caesarean delivery, from a rate of 1 in 2500 in the 1980s to as high as 1 in 533 deliveries today. Because of its increasing frequency, AS is now one of the most serious clinical challenges in obstetric practice [3, 4].

Variants of AS are basically classified into three categories according to the depth of trophoblastic growth: placenta accreta (villi are attached to the myometrium), placenta increta (villi invade the myometrium), and placenta percreta (PP; villi penetrate through the myometrium and to or through the serosa). PP is the leading cause of intractable postpartum haemorrhage due to its deep infiltration into the myometrium and the surrounding organs, such as the intestines and bladder [1].

Generally, the recommended management of suspected PP is a planned preterm caesarean hysterectomy to avoid massive maternal haemorrhage. However, this approach may not be considered the first-line treatment modality for women who desire future fertility. Therefore, surgical management of PP should be individualized to preserve fertility in appropriately selected cases [4].

In 2004, Palacios et al. described a uterus-preserving procedure for PP [5]. All patients included in their study had an anterior PP and, if possible, single incision (SI) via the trans-placental approach was performed at the upper border of the placental invasion area. If SI was not possible because of a widely invaded placenta, a second hysterotomy incision, double incision (DI), was performed for safe extraction of the foetus. Additionally, a few case reports [6–9] have been published about the conservative surgical approach for pregnancies complicated with PP.

In this study, we aimed to compare the effectiveness and results of DI and SI in the conservative management of PP.

## Methods

This was a retrospective cohort study with prospectively collected data conducted in the Prenatal Diagnosis Unit of a third level reference hospital, with approximately eighteen thousand deliveries each year. Twenty-two patients with PP who underwent conservative surgery between January 2014 and January 2016 were included in the study. The presence of PP was confirmed by histopathologic examination of all specimens.

This study was conducted according to the principles stated in the Declaration of Helsinki with subsequent revisions. The study protocol was approved by the Institutional Review Board of our hospital and was registered at clinicaltrials.gov (NCT 02702024).

Our clinical approaches to PP are preterm caesarean hysterectomy for patients with completed fertility and conservative management for patients who desire future fertility and meet the required criteria for conservative management, including uterus-preserving surgery. Our study population fulfilled the requisite criteria for conservative management. The potential risks and benefits of conservative surgery were explained to all study participants, and a written informed consent form was obtained. They were aware of the likelihood of hysterectomy based on the intraoperative findings.

### Antenatal surveillance

Routine antenatal examinations were performed. The gestational age was calculated from the first day of the last menstrual period and confirmed with a first trimester obstetric ultrasound.

Pre-diagnosis of PP was made by transvaginal and transabdominal ultrasonography with colour Doppler imaging. Placental mapping, including precise placental placement, upper placental borders, and placental findings, were recorded. Ultrasound findings indicating the probability of an increased invasive risk of PP are as follows:

1. Number of lacunae ≥ 3
2. Loss of retro-placental clear space or retroplacental myometrial thickness ≤ 1 mm
3. Loss of visualization of the myometrium and thickness of the myometrium, especially in the lower uterine segment with a previous caesarean scar
4. Bladder wall irregularity and interruption of the uterine wall and bladder interface
5. Increased sub-placental vascularity and turbulent blood flow extending from the placenta to the attached area and posterior bladder wall interface.

Patients with one or more of these risk factors were designated the high-risk of wide invasion group. The lowest myometrial thickness (retroplacental myometrium > 1 and ≤ 3 mm) and placenta lacunar spaces (<3 lacuna) were used to define the risk of low invasiveness in our patients, as previously described [10].

### Pre-operative planning

After the initial assessment, all patients were closely followed at two-week intervals until 34 weeks of gestation, which is when all patients were hospitalized. All elective surgeries were performed between 35 and 36 weeks of gestation. The assessment of the individual increased invasiveness risk of PP by ultrasonography was performed just prior to surgery in these patients. The formal pre-operative preparation for conservative surgery consisted of 4 units of packed red blood cells, 4 units of fresh frozen plasma, and 4 units of a thrombocyte suspension.

### Conservative surgery

All patients were placed in the dorsal lithotomy position during the surgeries. A three-way Foley catheter was inserted prior to the surgery. Neither ureteral stent placement nor arterial catheterization was used as a part of the management.

**Fig. 1:**
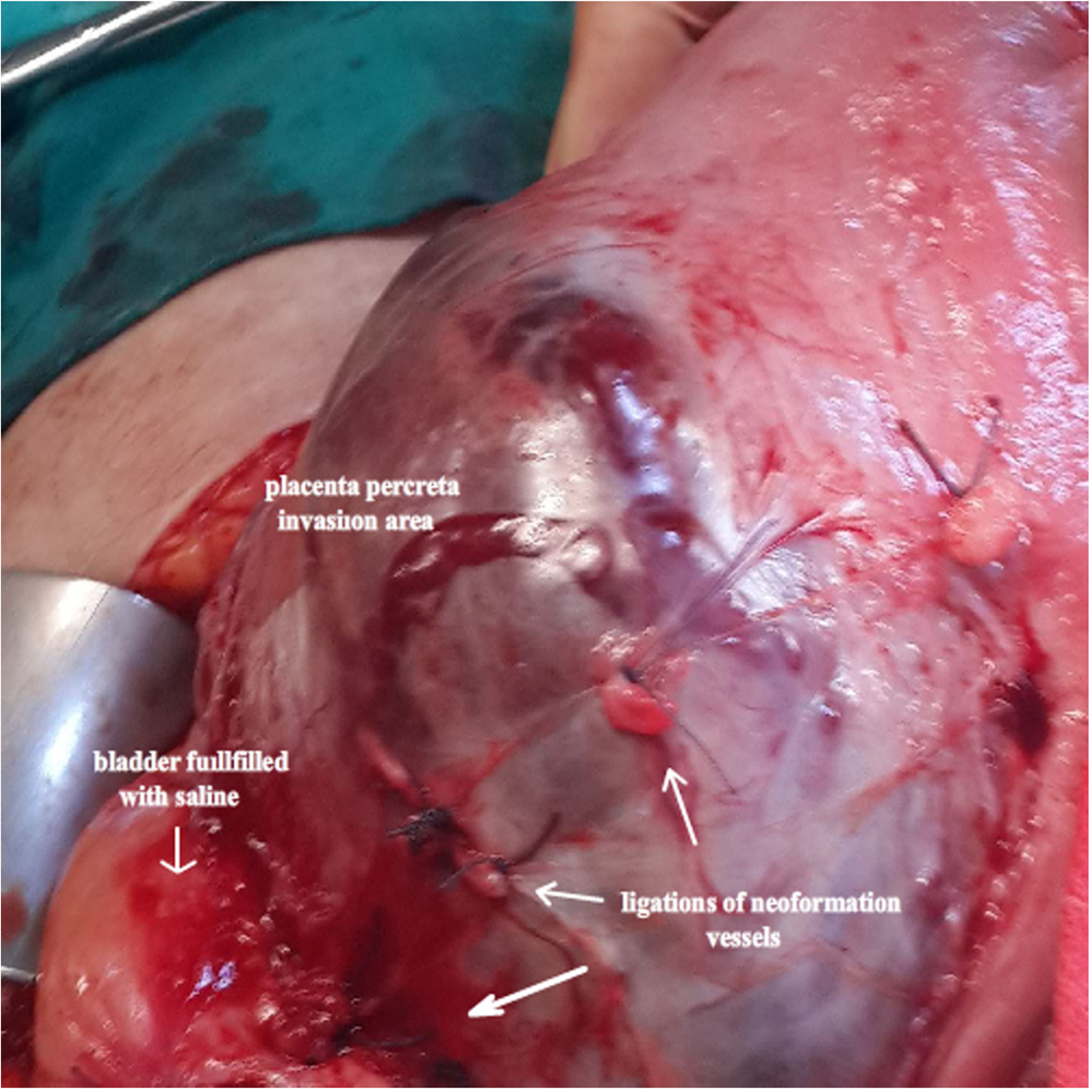
Dissection of involved bladder and ligation of neoformation vessels.

The individual risk of the patient for PP severity helped us choose the right type of abdominal skin incision. An infra-umbilical midline incision was made for the high-risk patients, and a Pfannenstiel incision was made for the low-risk patients.After laparotomy with either midline or transverse incision, the location of the placenta was examined to choose the type of uterine incision that was safe and optimal for both the mother and foetus. When the PP was not involved in the area of the previous uterine incision and the bladder dissection was considered feasible, the trans-placental segmental approach was preferred.Before dissection of the retro-vesical area, the bladder was filled with 200 cc of saline solution to clarify the borders of the bladder and neo-formation vessels between the uterine segment and bladder. During the dissection, ligations of neo-formation vessels were performed step by step (Fig. 1).

**Fig. 2:**
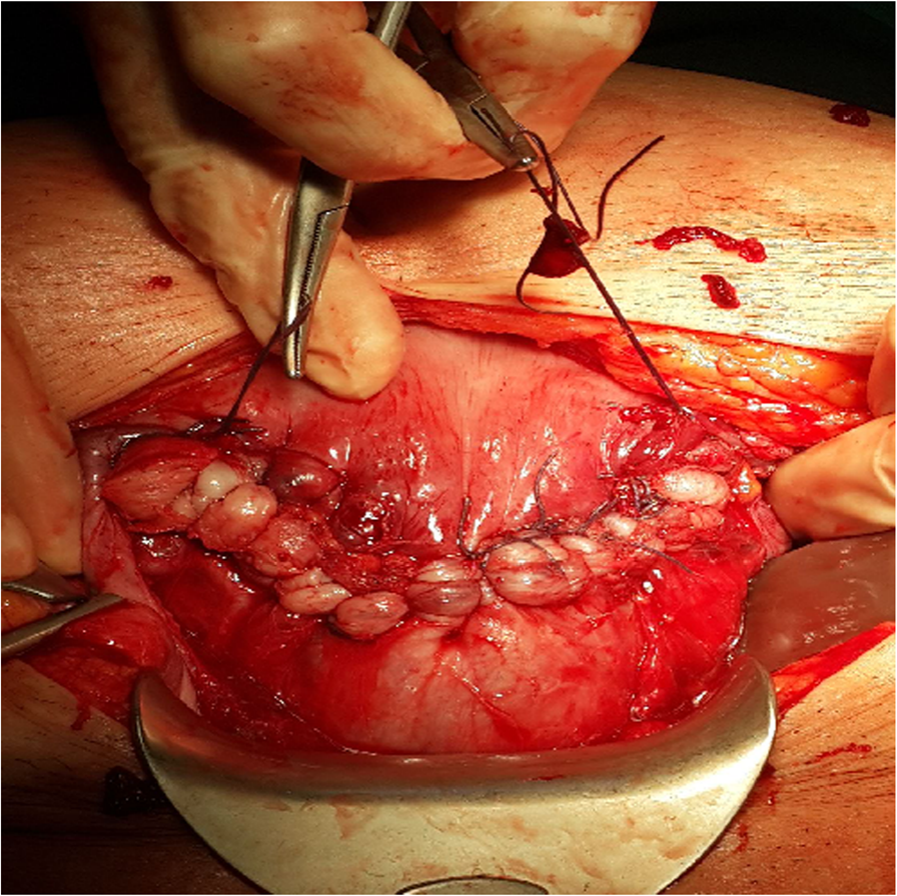
The segmental resection area closed with multiple Z-shaped sutures

Following the dissection of the retro-vesical space, hysterotomy was performed at the upper border of the involved segment. The myometrium with pathologic placental adherence was incised in the shape of an ellipsoid or wedge. The lower border of the incision was performed at least 2 cm above the isthmic part of the uterus (single incision technique for segmental resection). The segmental resection area was subsequently made, and the incision was entirely closed with multiple Z-shaped sutures (Fig. 2).

**Fig. 3:**
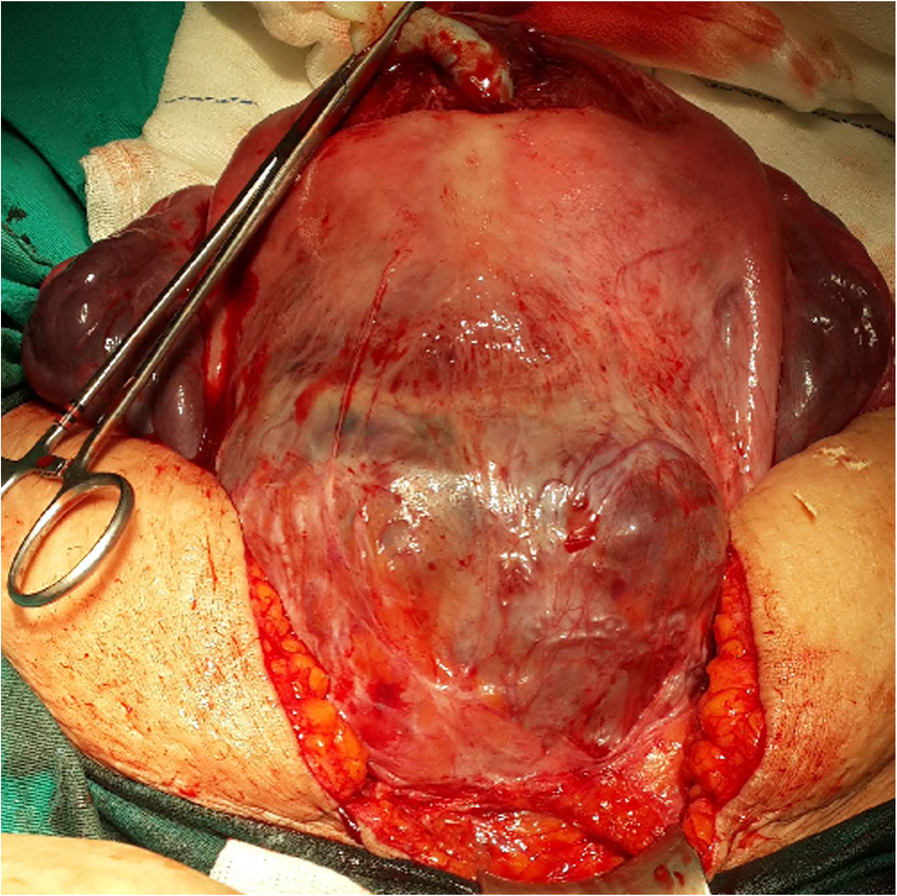
Foetal extraction with DI in widely invaded PP

**Fig. 4:**
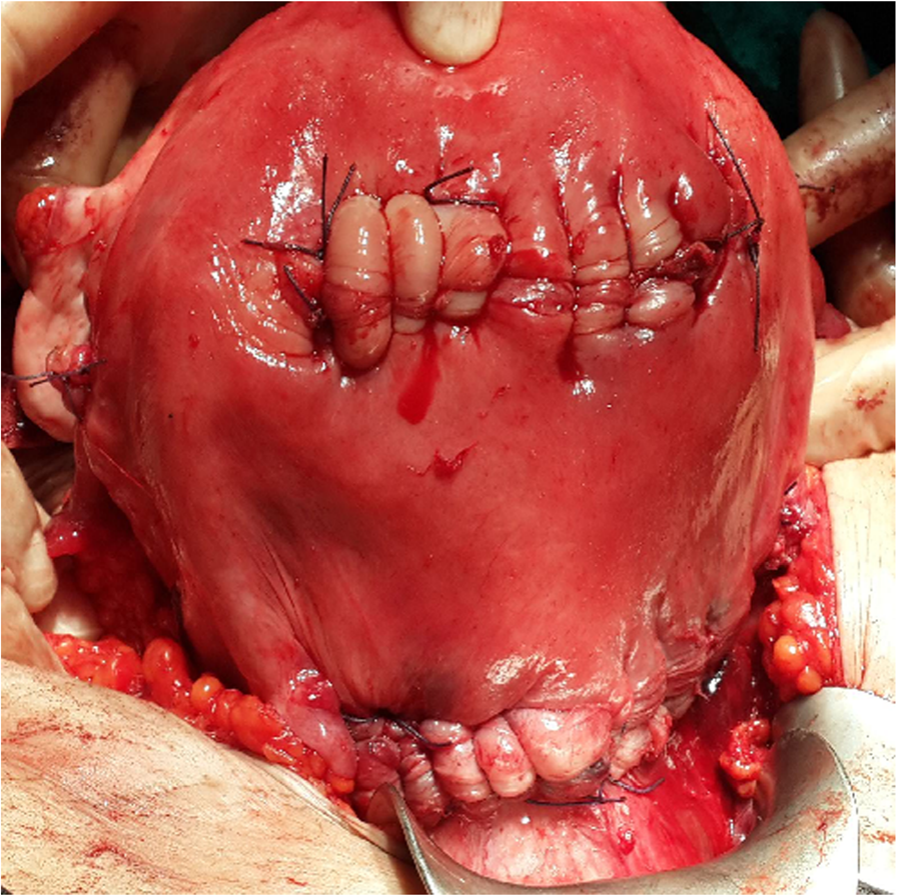
DI in uterus-preservation surgery for PP

However, the presence of a wider placental adhesion area did not allow for a trans-placental approach. In these cases, a transverse corporeal or vertical uterine incision was performed to avoid placental damage, and the foetus was immediately delivered through the uterine incision (Fig. 3). Following the extraction of the foetus and after opening the retro-vesical space, an ellipsoid or wedge incision was performed on the myometrium that did not including the pathologic placental adherence. As with the technique utilized for SI, the entire placental area was removed (double incision technique for segmental resection). The first incision used for foetal extraction was closed with single layer interrupted continuous sutures, and the segmental resection area was closed with multiple Z-shaped sutures in one piece (Fig. 4).

After closure of the uterine incision, a silicone drain was placed in the dissection area. We did not perform the ligation of large vessels before or during the surgeries in either technique. Twenty IU Oxytocin (Synptian Fort, Deva, Turkey) was intravenously administered after the delivery of the foetus and removal of the involved area.

In patients for whom segmental resection was technically impossible due to an extended invasion area, hysterectomy was the preferred mode of action to avoid uncontrolled haemorrhage.

### Post-operative follow up

The decision for intensive care unit admission was made by the anaesthesiologist. Post-operative patients requiring haemodynamic monitoring, ventilator support or extensive nursing care were admitted. All patients received thromboprophylaxis with 30 mg/day of enoxaparin (Clexane, Aventis Intercontinental, France). Utero-tonic drugs were continued within the first 24 hours.

**Fig. 5:**
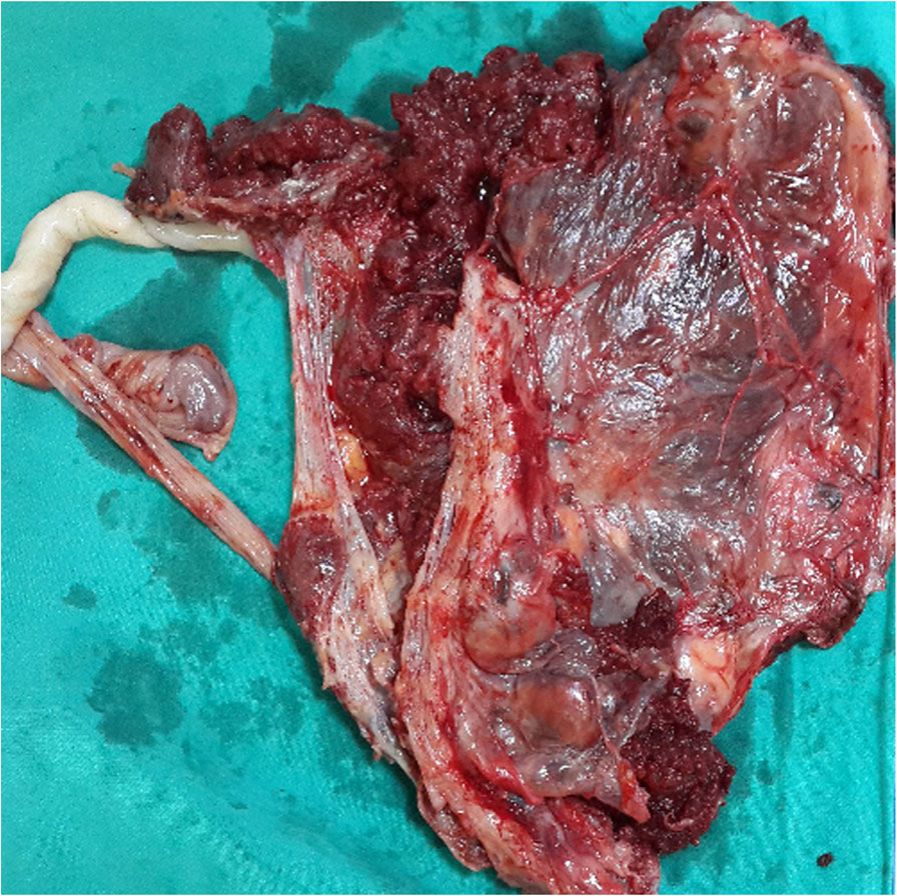
Histopathologic specimen of PP with a large invaded area

The histopathological specimens consisted of the excision of the whole placenta, myometrium and invaded surrounding tissue (Fig. 5). A diagnosis of PP was confirmed with histopathologic examination in all cases.

### Statistical analysis

Statistical analyses were performed using SPSS (Statistical Package for the Social Sciences) software (IBM SPSS 21.0 for Mac OS; SPSS Inc. Chicago, IL). Kolmogorov–Smirnov tests of normality were used to evaluate the distributions. Non-normally distributed variables were analysed with the Mann–Whitney U-test. Qualitative data were compared using Fisher’s exact test. Statistical significance was considered as *p* <0.05.

## Results

All the data for patients undergoing uterine preservation surgery for PP are shown in Table 1. Fifteen women underwent planned surgeries between weeks 35 and 36 of gestation, and seven women underwent emergency surgeries between weeks 29 and 39 of gestation. Of the 15 planned surgeries, 13 were performed through a midline incision, and two through a Pfannenstiel incision. Six of the seven emergency surgeries were performed with a Pfannenstiel incision.

**Table 1:** Patient characteristics

Patient no	Age	Gravida	Number of previous cesarean deliveries	Time of surgery	Gestational age at delivery	Type of skin incision	Type of second uterine incision	Number of uterin incision	Operation time (minutes)	Transfusion requirement (Total unit of packed red cells)	Intensive care unit admission (day)	Total hospitalization (day)
1	35	3	1	Elective	35	IUM	Transverse	2	90	0	1	4
2	30	3	2	Elective	36	IUM	Vertical	2	60	6	2	7
3	29	2	1	Elective	35	Pfan.	Transverse	2	60	0	0	3
4	29	3	2	Elective	36	IUM	Vertical	2	90	13	1	4
5	29	2	1	Elective	36	IUM	Transverse	1	60	4	0	3
6	33	6	1	Emergency	39	Pfan.	Transverse	1	60	2	0	3
7	25	4	3	Elective	35	IUM	Transverse	2	65	4	1	4
8	32	3	2	Emergency	38	Pfan.	Transverse	1	70	4	1	6
9	29	2	1	Emergency	38	IUM	Transverse	2	55	0	0	3
10	35	3	2	Emergency	29	Pfan.	Transverse	1	65	2	0	3
11	24	5	3	Elective	35	IUM	Transverse	2	70	2	0	4
12	30	4	3	Elective	36	Pfan.	Transverse	1	60	2	0	4
13	32	3	1	Elective	36	IUM	Vertical	2	70	4	0	4
14	23	2	1	Elective	35	Pfan.	Transverse	1	60	4	0	4
15	23	4	1	Elective	36	IUM	Transverse	2	65	4	0	3
16	28	3	0	Emergency	32	Pfan.	Transverse	1	55	0	0	3
17	24	3	2	Emergency	34	Pfan.	Transverse	1	45	0	0	3
18	28	4	3	Elective	35	Pfan.	Transverse	1	40	0	0	3
19	27	2	1	Elective	36	IUM	Transverse	1	70	2	0	3
20	29	5	2	Emergency	34	Pfan.	Transverse	2	50	0	0	3
21	22	3	1	Elective	35	IUM	Vertical	2	90	6	1	5
22	29	4	3	Elective	35	IUM	Transverse	2	45	0	0	3

*IUM* infra-umbilical midline, *Pfan*: Pfannenstiel

Of the twenty-two patients, segmental resection was performed via DI in 12 (54.55%), and the remaining 10 (45.45%) patients underwent segmental resection with SI. In the DI patients, the foetus was delivered via corporeal vertical incision in four (33.33%) patients and corporeal transverse incision in the remaining eight (88.9%) patients.

There were no complications in the SI group. In the DI group, conservative surgeries were successful in 11 (91.66%) patients. The remaining patient (Number 4) underwent re-laparotomy due to disrupted haemodynamics in the 15^th^ hour of the operation, with a suspicion of intra-abdominal bleeding. During re-laparotomy, approximately 1000 cc of bleeding from the bladder dissection area was found. Hysterectomy was performed due to the continuing leakage and the patient and relatives requesting definitive therapy before surgery.

Table 1 provides the comparison of the demographic characteristics of the patients for whom segmental resection was performed via SI or DI. There were no differences between the patients in terms of age, gravida, number of previous caesarean deliveries, gestational age at delivery, rate of elective surgeries and rate of transverse uterine incision. The rate of infra-umbilical midline skin incision was higher in patients with DI (88.89%) than in patients with SI (16.67%) (*p* = 0.003).

The comparison of the results of segmental resection via SI or DI is shown in Table 2. There were no differences between the groups in terms of the operation time, transfusion requirement, intensive care unit admission, total hospitalization or success of conservative surgery (Table 3).

**Table 2:** Comparison of the demographic characteristics of patients for whom segmental resection was performed via single or double uterine incision

	Single uterine incision (*n* = 10)	Double uterine incision (*n* = 12)	*p* value
Age	28.5 (29–35)	29 (22–35)	0.739
Gravida	3 (2–6)	3 (2–5)	0.489
Number of previous caesarean deliveries	1.5 (0–3)	1.5 (1–3)	0.751
Gestational age at delivery (weeks)	35.5 (29–36)	35 (34–38)	0.945
Elective surgeries	5 (50%)	10 (83.33%)	0.095
Skin incision with IUM	2 (20%)	10 (83.33%)	0.003

*IUM* Infra-umbilical midline incision, continuous variables are given as the medians (ranges: minimum and maximum) and categorical variables are given as the number (percentages)

**Table 3:** Comparison of the segmental resection results for single and double uterine incision

	Single uterine incision (*n* = 10)	Double uterine incision (*n* = 12)	p value
Operation time (minutes)	60 (45–70)	65 (45–90)	0.203
Used blood and blood products (total units)	2 (0–4)	3 (0–13)	0.628
Intensive care unit admission (days)	0 (0–1)	0 (0–2)	0.098
Total hospitalization (days)	3 (3–6)	4 (3–7)	0.226
The success of conservative surgery	10 (100%)	11 (91.67%)	0.35

Continuous variables are given as the medians (ranges: minimum and maximum), and categorical variables are given as the number (percentages)

## Discussion

In cases of PP where the placenta covers the lower uterine segment, it may be necessary to cross the placenta to deliver the baby. In addition, due to the poor contractility of the lower uterine segment and because of the increased blood supply, this manoeuvre usually produces additional haemorrhage. If the bleeding is not controlled, the process may be aggravated and end in coagulopathy or other severe complications [11]. On the other hand, in this serious condition, it is necessary to deliver the baby quickly and control the haemorrhage to avoid shock and coagulopathy. This is not a minor issue, especially when the response time is short [12].

There is no current universal treatment for PP. Management could be different according to personal or maternal preferences and medical experiences, skills and resources. Although, several approaches are available, all aim to avoid maternal bleeding during delivery [13]. PP can be managed conservatively without hysterectomy in the following two ways: (i) leave the placenta in situ and (ii) partial myometrial resection of the entire placenta and restoration of the uterine anatomy. Each method has weaknesses and strengths. The major advantage of myometrial resection is that it allows for pathological confirmation of PP [12].

Palacios et al. published the largest series on the conservative treatment of PP worldwide [5]. A uterine incision was usually located in the upper section of the involved segment, and the trans-placental approach was recommended, when possible. Haemostasis was achieved with additional surgical procedures, such as selective vascular ligatures of the large vessels, and additional coagulant materials, such as fibrin glue and polyglycolic mesh. They performed an antero-posterior fundal extra hysterotomy (DI) to extract the foetus when segmental dissection was not feasible due to tissue fusion of the involved areas. The success rate was 50/68 (73.53%) in this case series. Our study is the first to compare DI and SI via the trans-placental approach in uterus-preserving surgery for PP.

### DI was based on the following three aims:

1. Safe extraction of the foetus, decreasing time loss during vesico-uterine dissection and damage to the foetal-placental circulation
2. Reduce blood loss due to the incision adjacent to pathologically adhesive placenta
3. Easily change the surgery type to hysterectomy, if necessary.

The dissection of the vesical-uterine fold should be performed before extraction of the foetus in SI. Bladder invasion is a severe complication of PP. Abnormal blood supply of invaded tissue, adhesions, a narrow operating space and a lack of dissection planes may make this surgery exceedingly difficult for the surgeon. The dissection is processed step by step with ligation through neoformation of vessels and might take a long time and substantial effort. The DI technique first aims to protect the foetus from the time loss during vesical-uterine dissection. Because of the propensity for severe haemorrhage, almost all patients with PP undergo general anaesthesia. In a previous study, it was shown that induction-to-delivery intervals of more than eight minutes and uterine incision-to-delivery intervals of more than three minutes were associated with a greater incidence of low Apgar scores and significantly more instances of neonatal acidosis [14]. The severity of adhesion is usually established when attempts are made to separate the adherent placenta from the bladder. This manoeuvre can cause massive haemorrhage that is often quite challenging to control [15]. The best procedure must protect the foetus from massive haemorrhage and avoidable complications during delivery. The second incision is far from invasive, and adhered placenta could protect the foetus from the negative effects of massive bleeding due to the dissection. Extraction of the foetus might enhance vision in the area of the operation. Moreover, the surgeon could more calmly concentrate on the placenta dissection. Caesarean hysterectomy with a PP in situ is clearly more difficult than other elective caesarean hysterectomies [16]. There is a greater need to both maintain a margin from the vascular cervical-placental mass and protect the ureters and vesical. The region of PP will most often involve the lower uterine segment and mid posterior fundus of the bladder. It is important that the uterus is devascularized as much as possible before this region is approached [17]. DI should give surgeons sufficient time for segmental resection, and all of these interventions might be performed to prevent massive haemorrhage.

We compared results of segmental resection via SI or DI. The characteristics of the cases, such as the ages, gravidas, numbers of previous caesarean deliveries, and gestational ages on delivery, were similar. Five of the 10 cases in the SI group underwent operations in emergency conditions, and the skin incision was the Pfannenstiel incision in four of these cases. Only two cases with DI had surgeries in emergency conditions. An infra-umbilical midline incision was markedly more common in DI (10 of 12) compared to in SI (two of 10 cases). We chose the type of skin incision because of the individual risk of cases. In cases with a high risk for wide invasion, which had anterior placental placement and dense, widespread invasion, the skin incision was planned as an infra-umbilical midline incision. This method prevented unnecessary infra-umbilical midline incisions. These findings support our success with pre-operative foresight. In high-risk patients, we preferred an infra-umbilical midline incision before surgery, and these patients underwent segmental resection with DI after laparotomy. On the other hand, there might have been a potential selection bias in our study because it was retrospectively designed, even though the data were prospectively collected.

Although DI was applied to patients who had a high risk of wide invasion, we have seen that DI did not extend the operation time. No study has discussed blood loss or the need for transfusion based on focusing on PP cases who were treated conservatively. Wright et al. examined predictors of blood loss for women with placenta accreta who had undergone hysterectomy [18]. The median estimated blood loss was three litres, and the mean transfusion requirement was five units. In our study, the median transfusion requirements were three units in the SI group and four units in the DI group. There was no statistically significant difference. A patient underwent re-laparotomy in response to disrupted haemodynamics in the 15^th^ hour of operation, with suspicion of intra-abdominal bleeding, and received 13 units of packed red cells. When we excluded this case, the median transfusion requirement was 2 units. SI or DI did not change the number of days spent in the intensive care unit or hospital. Additionally, the success rate of conservative surgery was not different between SI and DI.

Reported complications in the conservative surgery of PP included venous haemorrhage, rupture of an epigastric artery and disseminated intravascular coagulation. Ureteral lesions, simple vesical fistula, iatrogenic foreign body and uterine infection with anaerobic gram-negative bacteria were reported in previous studies [5, 19]. We observed mild disseminated intravascular coagulation in one patient. This patient underwent re-operation, and we performed hysterectomy. We have not observed any vesical injury in our case series. We thought that this could be due to the effect of filling the bladder before dissection of the retro-vesical space. This method helps the surgeon identify the borders of the bladder and neoformation vessels. However, this issue requires support from further studies. There was a urinary system infection in one patient in each group, and the patients were treated with appropriate antibiotics.

Long-term complications are a constant source of concern in the conservative management of PP. Palacios et al. reported on 10 pregnancies in 42 patients who were followed up for 3 years after surgery [5]. They did not observe any complications during the pregnancies, and vesical and segmental dissection were easily performed in all of them. DI could improve the risk of uterine rupture; however, this hypothesis needs to be tested with long-term follow-up studies. It has been reported that there is an approximate 22-28% recurrence risk of PP when patients undergo conservative management and have a subsequent pregnancy [20, 21]. This risk should be discussed with women who have an antenatal diagnosis of PP and who may be considering conservative treatment.

We acknowledge the limitations of this type of case series report and the susceptibility to bias. However, it is clinically challenging to perform a study with large cohorts on such a rare condition.

## Conclusions

Based on the outcomes of our study, DI and SI seem to be safe surgical approaches for delivering the foetus and preventing severe haemorrhage. DI could allow for safe extraction of the foetus from this chaotic condition without worsening the results of SI in uterus-preserving surgery for PP. Pre-operative ultrasound findings and clinical experience guide the type of skin incision that adequately supports the chosen surgical procedure.

PP is one of the leading causes of postpartum haemorrhage, and designing a prospective randomized trial on PP is difficult because of its rarity and ethical concerns. Observational or retrospective studies could provide useful insights into the best treatment approach. It would be better to study these techniques in many patients to evaluate their safety, efficacy, and long-term outcomes.

## Abbreviations

PP: Placenta percreta
DI: Double uterine incision
SI: Single uterine incision
